# On the Design of Thermal-Aware Duty-Cycle MAC Protocol for IoT Healthcare

**DOI:** 10.3390/s20051243

**Published:** 2020-02-25

**Authors:** Muhammad Mostafa Monowar, Madini O. Alassafi

**Affiliations:** Department of Information Technology, Faculty of Computing and Information Technology, King AbdulAziz University, Jeddah 21589, Saudi Arabia; malasafi@kau.edu.sa

**Keywords:** wireless body area networks, duty cycle MAC, thermal-aware

## Abstract

Wireless Body Area Networks (WBANs) are designed to provide connectivity among diverse miniature body sensors that support different Internet of Things (IoT) healthcare applications. Among diverse body sensors, WBANs exploit in-vivo sensor nodes that detect and collect the required biometric data of certain physiological change inside the human body, and transmits the sensed data utilizing wireless communication. However, sensing and wireless communication activities of in-vivo sensors produce heat and could result thermal damage to the human tissue if the sensing and communication continues for a long period. Furthermore, Quality of Service (QoS) provisioning for diverse traffic types is another striking requirement for WBANs. These pressing yet conflicting concerns trigger the design of ThMAC—a Thermal aware duty cycle MAC protocol for IoT healthcare. The protocol regulates the communication operation of a body sensor based on estimated temperature surrounding a tissue to maintain moderate temperature level in a body, also avoiding hotspot. Exploiting both contention-based and contention free channel access mechanisms, ThMAC introduces a superframe structure, where disjoint periods are allocated for diverse traffic types to achieve QoS provisioning. Moreover, ThMAC ensures a reliable and timely delivery of sporadically generated emergency data through an emergency data management mechanism. ThMAC performance is evaluated through computer simulations in terms of thermal rise, energy consumption as well as QoS metrics such as delay and reliability. The results show superior performance of ThMAC compared to that of IEEE 802.15.6.

## 1. Introduction

The evolution of fifth generation (5G) wireless communication technologies has reached an unprecedented height with the aim of providing connectivity for any device type and any application. All of these potential applications exploiting 5G come under the name of Internet of Things (IoT). Thanks to the Moore’s law, the continuous miniaturization of electronic devices [1] whilst growing their capacity eventually led to the development of tiny and portable sensors, capable of communicating wirelessly which form the basis of IoT. Among many other applications, IoT healthcare is one of the prominent ones and gained tremendous interest to the research community [2].

Wireless Body Area Networks (WBANs) is a network paradigm designed to provide connectivity among diverse miniature body sensors that support different IoT healthcare applications. Wireless Body Area Networks emerge as a promising solution to threat the ever-increasing healthcare expenses [3,4]. Although Wireless Sensor Networks (WSNs) continue to develop for a wide range of applications, they cannot precisely address the challenges of human body monitoring. Human body monitoring can be achieved through attaching sensors to the body surface in conjunction with embedding them inside the tissues to collect more factual physiological data for the healthcare professionals. This will considerably reduce the overall health care cost, yielding better utilization of healthcare resources. This also brings about other opportunities including infant monitoring as well as independent living of aged people.

A WBAN can thus be equipped with implanted or wearable sensors capable of sensing and transmitting body parameters from diverse body locations [5]. However, the tiny sensors are required to transmit their data to a more capable device, known as Body Coordinator (BC) for furtherprocessing and communicating with the users. The continuous sensing as well as communication of the sensed parameters; however, produce heat resulting in severe thermal damage to the human tissue if it prolongs for significant period of time [6,7,8] which also might eventually appear as a threat to human life. Therefore, thermal-rise is regarded as one of the crucial factors while designing communication protocols for WBANs.

Due to the high potential of a WBAN having in-vivo sensors, it can be utilized to measure a wide range of body parameters including temperature, blood pressure, blood oxygen saturation, pulse, electrocardiogram (ECG) and so forth [9]. The diverse sensing parameters also possess distinct Quality of Service (QoS) requirements in terms of delay and reliability. For instance, electroencephalogram (EEG), electrocardiogram (ECG) and electromyography (EMG) and so forth require timely delivery while respiration monitoring, pH level monitoring has reliability constraint. Furthermore, due to potentially life threatening situations, some parameters require emergency transmission with reliability. The in-vivo sensors are also typically powered by battery having limited energy capacity. Hence, low energy consumption is another vital requirement for WBAN communication protocols to prolong the network lifetime.

The MAC protocol for a WBAN mainly coordinates the channel access to avoid collisions and maximize the throughput. However, in a highly constrained WBAN with a number of conflicting objectives, particularly maintaining lower thermal rise and energy consumption as well as meeting the respective QoS demands of diverse traffic types is a profoundly challenging task. A good number of studies attempted to devise MAC protocol for WBAN [10,11,12,13,14,15,16,17,18,19,20,21,22,23]. The majority of them focused mainly on energy efficiency and QoS provisioning. No significant research exists addressing the thermal-rise issue of WBAN except in Reference [24]. Here, the authors proposed temperature aware probabilistic sleep cycle management for WBANs. However, the study mainly investigates the effect of different probabilistic sleep duration on thermal rise and attained throughput rather than devising a MAC protocol.

To address the aforementioned challenges, we propose a Thermal-aware duty cycle MAC protocol (ThMAC) for IoT healthcare. We intend to present a full-fledged MAC scheme, which on the one hand controls the duty cycle of the sensor nodes aiming to reduce thermal rise of the body sensor nodes, and on the other hand attempts to deliver the diverse physiological parameters meeting their respective QoS demands. Overall, our contributions are as follows:

- We present a thermal-aware wake-up schedule mechanism with the aim of reducing thermal-increase as well as avoiding the creation of hotspot.
- We provide a traffic taxonomy considering the diverse QoS demands.
- We introduce a superframe structure and designate distinct transmission periods exploiting both contention based and contention free medium access for diverse traffic types aiming to reduce contention along with treating the traffic based on their QoS requirements. Additionally, small and big DATA are treated differently to achieve fair QoS provisioning.
- We introduce an Emergency Data Management scheme with an intent to deliver urgent traffic reliably and timely.
- We perform extensive simulations to evaluate the performance of ThMAC and prove its effectiveness.

The rest of the paper is organized as follows: Section 2 summarizes the related works. Section 3 presents some preliminaries and assumptions behind the protocol. Section 4 describes the protocol operations in detail. Section 5 demonstrates the protocol performance using simulation. Finally, Section 6 presents the concluding remarks.

## 2. Related Works

A good number of studies are found in the literature that introduced MAC protocols for WBANs. IEEE 802.15.6 was standardized for WBAN in 2012, with specifications provided for PHY and MAC layers [10,25]. The standard exploited only star topology, where nodes deployed on/in the human body are coordinated by a hub. IEEE 802.15.6 adopted a hybrid approach combining both contention based CSMA/CA as well as contention free TDMA like scheme. The superframe is divided into a number of periods including Exclusive Access Phases (EAP1 and EAP2), Random Access Phases (RAP1 and RAP2), a Managed Access Phase (MAP) and a Contention Access Phase (CAP). The EAPs are utilized for higher priority or emergency traffic, the RAPs are used for transmitted non recurring traffic. The MAP usually uses TDMA to support contention free transmission such as polling and scheduled allocation. The standard defined a number of User Priorities (UPs) for diverse traffic types ranging from 0 to 7. The priority value 0 is regarded as the lowest priority while the priority value 7 is the highest priority. For differentiated service provisioning, different contention values are specified for diverse traffic priorities. The standard stimulated the rapid development of WBAN and there has been an upsurge of research on WBAN in recent years.

In Reference [11], the authors proposed an adaptive MAC protocol based on IEEE 802.15.6. This study dynamically adjusted the length of contention access and non-contention access phase based on the proportion of nodes that generate priority data. B. Liu et al. [12] introduced a context-aware MAC protocol satisfying time varying demands of WBAN based on channel status and traffic nature. In Reference [13], the authors introduced an efficient and energy efficient MAC protocol, HE-MAC to increase network reliability and reduce energy consumption. Exploiting TDMA, an adaptive MAC protocol is proposed in Reference [14] that defined a synchronization scheme to avoid collision. H. Li et al. introduced a heatbeat driven MAC protocol (H-MAC) for WBANs [15]. H-MAC exploits the heartbeat vibration from physiological signal with some sensors as synchronization ticks to avoid any time synchronization required in TDMA based MAC protocol.

Some studies also focused on only emergency traffic transmission. Jaeho Lee et al. proposed an emergency prioritized asymmetric protocol for WBANs [16]. This study attempted to provide diverse energy balance between nodes and the coordinator yet ensuring the timely delivery of emergency data. Following the same objective, MEB-MAC has been proposed [17] that balances the conflicting requirements of energy balance and QoS provisioning. A priority based MAC protocol is introduced in Reference [18] that employed a contention free period for emergency traffic transmission and a contention access period for regular periodic traffic transmission. Zhang et al. proposed MEM-MAC—an energy efficient MAC for medical emergency monitoring body sensor networks [19]. MEM-MAC is designed assuming the infrequent occurrence of emergency traffic and tries to balance between energy efficiency as well as packet delivery latency.

Some earlier efforts [20,21,22,23] are also found exploiting the previous IEEE 802.15.4 standard [26], which was basically designed for the typical WSNs. These protocols modified the IEEE 802.15.4 structure to achieve mainly energy efficiency and QoS provisioning to some extent.

Considering the effect of thermal rise in a body, a temperature aware probabilistic sleep cycle management scheme has been proposed for WBANs [24]. This study examined three random sleep durations including LogNormal, Poisson and Binomial distributions, and observed the effect of overall temperature rise of the nodes and the achieved throughput. However, the study did not reflect the effect of actually measured temperature on the sleep duration of the nodes that might cause some highly heated nodes to be participated in communication activities. Moreover, the study only focused on observing the thermal effect through random sleep duration rather devising a full-fledged MAC scheme.

According to the aforementioned research background, it is obvious that the existing efforts for developing MAC for WBANs mainly focused on energy efficiency and QoS provisioning. To the best of our knowledge, no solution exists that devised a comprehensive MAC scheme taking into account the thermal effect of in-vivo sensor nodes in addition to achieve QoS provisioning and emergency data handling. These motivate us to devise ThMAC.

## 3. Preliminaries and Assumptions

This section presents the network model we considered along with some assumptions, as well as the traffic taxonomy.

### 3.1. System Model

**Figure 1:**
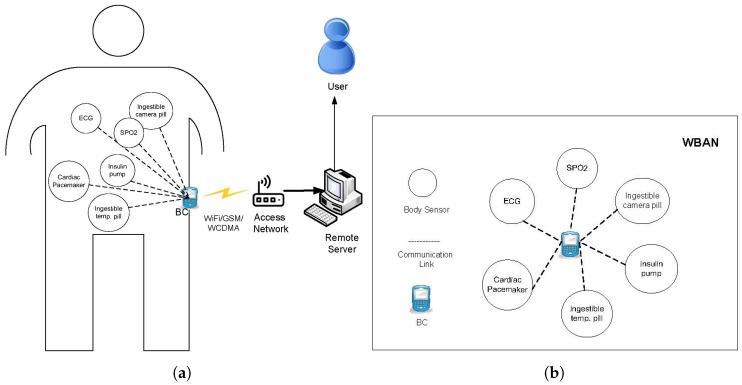
Network Model.

We consider a deployment scenario, in which heterogeneous in-vivo nodes are implanted in a human body forming a WBAN. A central data sink, also known as Body Coordinator (BC) is attached to the body surface as shown in Figure 1a. The BC is not power hungry and could be equipped with external power source, however, the in-vivo nodes are usually energy constrained. In-vivo sensor nodes mainly perform sensing and communication activities while the BC manages some major operations of WBAN such as data aggregation, synchronization, context recognition and exchange of control and management packets. The BC accumulates the data from the in-vivo nodes, process it and then transmits it to a remote server through access networks using WiFi, GSM or WCDMA as depicted in Figure 1a, and this communication paradigm is out of the scope of this paper.

The above deployment scenario forms a star topology as shown in Figure 1b, where the body sensors are directly connected to he BC. We denote a body sensor node as $n_{i}$, and assume each node $n_{i}$ communicates with the BC using fixed transmission power. We further assume the communication links between a $n_{i}$ and BC are symmetric.

### 3.2. Traffic Taxonomy

In an implanted WBAN, the traffic generated by the in-vivo body sensors are inherently diverse in regard to their Quality of Service (QoS) demands. Considering the diverse QoS requirements, we categorize the traffic as follows:

- *Emergency (Em)* traffic: The traffic of this category possesses stringent delay and reliability requirements, and thus to be reported urgently whenever a node senses some unusual variations in physiological parameters.
- *Delay constrained (Dc)* traffic: This traffic category requires being delivered with lower delay, although it allows packet losses to some extent, for instance, telemedicine video streaming application.
- *Reliability constrained (Rc)* traffic: The traffic belonging to this type has high reliability requirements but it can tolerate some delay. Respiration monitoring, pH-level monitoring etc. are some examples of reliability constrained traffic which can be processed offline, but packet losses may cause a drastic effect.
- *Normal (Nr)* traffic: This traffic category does not have a strict delay nor reliability constraints. Periodic vital sign monitoring applications generate normal traffic when the sensed value falls below some threshold level.

## 4. ThMAC Design

The main goal of the proposed ThMAC is to reduce thermal increase due to wireless communication of the in-vivo sensor nodes as well as to avoid the creation of a hotspot. Furthermore, we opt to meet the diverse QoS requirements of different traffic types in terms of delay and reliability. Considering the energy constraints of in-vivo sensor nodes, ThMAC aims to reduce the energy depletion of the nodes to prolong the network lifetime. Finally, ThMAC also addresses emergency traffic handling and tries to deliver the Em traffic to the least possible delay with high reliability. This section discusses the design details of ThMAC.

### 4.1. Modeling Thermal Rise

ThMAC adopts the model for thermal rise similar to the model as presented in Reference [7,8]. This model assumes the WBAN or the part of it is inside the cross section of a tissue, and the cross section is further divided into grids. An in-vivo node is assumed to be located in a grid of fixed length and width. Each node in a grid is assumed to have an initial temperature which gradually varies due to the sensor communication.

One of the root causes for thermal increase is the radiation from the node antenna. To estimate the radiation level absorbed by the tissue, Specific absorption rate (SAR) is exploited. The space surrounding the antenna is split into near and far field. SAR in the near and far field can be estimated as follows [7]:

(1) $$SAR_{NF}=\frac{\sigma \mu \omega }{\rho \sqrt{\sigma^{2}+\epsilon^{2}\omega^{2}}}{\left(\frac{Idlsin\theta e^{-aR}}{4\pi }\left(\frac{1}{R^{2}}+\frac{\gamma }{R}\right)\right)}^{2}$$

(2) $$SAR_{FF}=\frac{\sigma }{\rho }{\left(\frac{{\bar{a}}^{2}+{\bar{b}}^{2}}{\sqrt{\sigma^{2}+\omega^{2}\epsilon^{2}}}\frac{Idl}{4\pi }\right)}^{2}\frac{sin^{2}\theta e^{-2\alpha R}}{R^{2}},$$

where, *R* denotes the distance between the source and the observation point, γ is the propagation constant, dl is the conducting wire length for a short dipole antenna, σ is the medium conductivity, *I* is the amount of current, ϵ is the relative permittivity, μ is the permeability, ρ is the mass density and sinθ=1.

Another factor that contributes to thermal rise [7] is the power dissipation of the sensor node circuitry. This power dissipation can be computed as power dissipation density, $P_{c}$, which is estimated through dividing the power consumed by the sensor circuitry by the sensor volume.

Taking into account these two causes for thermal rise, the temperature of a node at a grid point (x,y) at time *t*, denoted as $T^{t}\left(x,y\right)$, can be estimated utilizing FDTD [27] which is an electromagnetic modeling technique that discretizes the differential form of space and time [7].

(3) $$\begin{array}{c} T^{t}\left(x,y\right)=\left(1-\frac{\Delta_{t}b}{\rho C_{p}}-\frac{4\Delta_{t}K}{\rho C_{p}\Delta^{2}}\right)T^{t-1}\left(x,y\right)+\frac{\Delta_{t}}{C_{p}}SAR\\ \;+\frac{\Delta_{t}b}{\rho C_{p}}T_{b}+\frac{\Delta }{\rho C_{p}}P_{c}+\frac{\Delta_{t}K}{\rho C_{p}\Delta^{2}}(T^{t-1}\left(x+1,y\right)\\ \;+T^{t-1}\left(x,y+1\right)+T^{t-1}\left(x-1,y\right)+T^{t-1}\left(x,y-1\right)). \end{array}$$

Here, $\Delta_{t}$ is the discretized time step, Δ is the discretized space step, *b* is the blood pressure perfusion constant, $C_{p}$ is the specific heat of the tissue, $T_{b}$ is the fixed blood temperature, *K* is the thermal conductivity of the tissue.

From Equation (3), we can find the temperature of a node at grid point (x,y) at time *t* which is a function of the temperature at (x,y) at time t−1, and the function of the temperature of surrounding nodes at grid points ((x+1,y), (x,y+1), (x−1,y), and (x,y−1)). The assumption for fixed node positions is valid since the in-vivo nodes are surgically implanted. If we know the tissue properties, the properties of blood flow, and the heat absorbed by the tissue, we can easily estimate the temperature at a given time.

### 4.2. Superframe Structure

**Figure 2:**
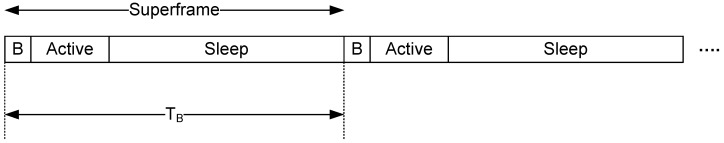
Basic Superframe structure of ThMAC.

Figure 2 illustrates the basic superframe structure of ThMAC. ThMAC adopts the beacon enabled mode operation due to its flexibility to manage and regulate the superframe structure. The superframe starts with a beacon period where BC broadcasts a beacon frame containing management information which includes initial synchronization with the body sensors such as clock information, superframe length, active period length, duty-cycle information etc. The beacon frame is generated with relatively long interval, denoted as $T_{B}$, also known as superframe period.

**Figure 3:**
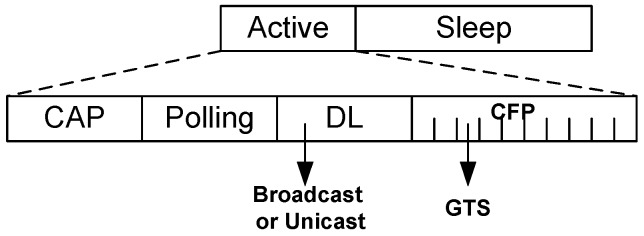
Active period structure.

Following the beacon period, active period starts which is further divided into Contention Access Period (CAP), Polling period, Download Period (DL) and Contention Free Period (CFP) as depicted in Figure 3. During CAP, nodes employ random access protocol, for example, CSMA/CA and transmits sensed data to the BC. The polling period exercises the controlled access where the BC deliberately polls certain body sensor nodes to upload their data which also ensures reliable transmission. The DL period is used for transmitting data in the form of broadcast or unicast from the BC to the nodes. CFP, followed by DL contains some guaranteed time slots (GTS) to ensure contention free reliable transmission for certain nodes. The slot assignment of the nodes will be notified through a broadcast packet during DL period.

Being a duty cycle MAC protocol, ThMAC employs a long sleep period where the nodes enter to the deep sleep condition or low power listening mode for energy conservation. Usually no transmission is allowed during sleep period but we grant an exception for emergency data transmission as to be discussed later.

**Figure 4:**
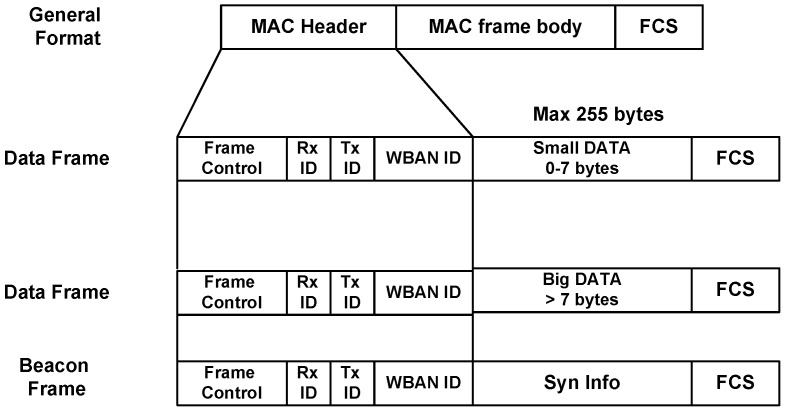
MAC frame format of ThMAC.

One of the striking design goals of ThMAC is QoS provisioning of diverse body sensor types. Considering the respective QoS demands, we exploit different periods of an active period for the transmission of diverse traffic. CAP is designated for Dc and Nr traffic transmission. However, due to the delay constraint, Dc exploits prioritized transmission as to be discussed in Section 4.4.1. The polling period mainly handles Rc traffic being reliability constrained. Both CAP and Polling period are utilized for small DATA transmission. However, nodes transmit the big DATA frame during CFP period. Figure 4 portrays the frame structure of ThMAC. In a WBAN, most of the traffic are of periodic nature, and small payload length of 7 bytes can accommodate for most commands and data, and we term this frame as small DATA frame. The payload length exceeding 7 bytes is regarded as big DATA frame. In a superframe, if a node generates big DATA of either Dc or Rc traffic type, it requests for the desired number of GTS slots during CAP or Polling period. Upon receiving the request, BC allocates the required GTS slots for the big DATA and notify them during DL period.

The main benefit for the allocation of diverse periods for diverse traffic types lies in reducing the contention that could result in lower latency and higher packet delivery ratio. Moreover, since the occurrence of big DATA is not common in nature, designating a separate CFP period ensures the fairness with the frequent small DATA in terms of latency, reliability as well as energy consumption.

In a WBAN, emergency traffic is treated as the most critical one that needs to be reported urgently. Em traffic usually occurs when a node senses some unusual variations in physiological parameters that might happen any time during the superframe. Therefore, ThMAC does not bound the Em traffic transmission into a certain period rather than allowing it in any period (i.e., CAP, Polling, CFP and even sleep period).

### 4.3. Thermal Aware Wake-Up Schedule

This section presents a thermal-aware wake-up schedule mechanism with the aim of reducing thermal-increase as well as avoiding the creation of a hotspot.

As mentioned earlier, the main reason for thermal increase around the tissue is the radiation from the node antenna, which depends on the degree of communication activities by the sensor nodes. In particular, the continuous operation of a node’s transceiver raises the temperature of the node surrounding a tissue and eventually reaches to a certain level causing tissue damage. We define that threshold level of the temperature as the hotspot threshold, denoted as $Th_{h}$. In contrast, the lack of communication operation gradually causes a node to be cooled down. Therefore, by controlling the communication activity the temperature around a node can be regulated.

**Figure 5:**
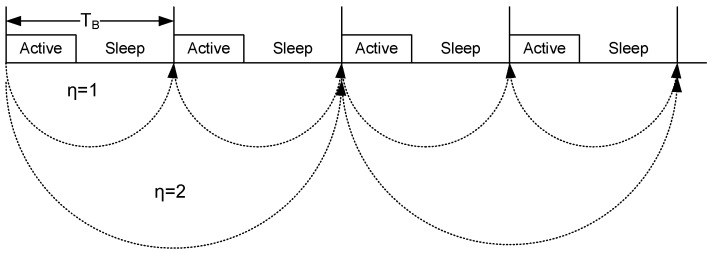
Communication Period.

The basic idea of our proposed scheme is as follows. To control the communication activity of a node, we define *communication period*, η which is the superframe interval where nodes communicate with the BC. For instance, for a node $n_{i}$, if η=1, then the node communicates with the BC in every superframe. A node communicates with the BC in every second superframe for η=2 (Figure 5). Hence, for η=m, frames are exchanged every $m^{th}$ superframe between the node and the BC, and the node is allowed to sleep between *m* superframes. Our thermal aware wake-up schedule scheme governs the value of η, to regulate the temperature around the sensor.

Algorithm 1 presents the thermal aware wake-up procedure of ThMAC. We adopt the multiplicative increase and additive decrease (MIAD) to regulate η. Here, we define four constant parameters: maxη, minη, α and β. maxη and minη refer to the maximum and minimum value of communication period η respectively. We empirically set these parameters as maxη=8 and minη=1. α and β are the well known increasing and decreasing factors for MIAD algorithm, and here we choose α=2 and β=1.

**Algorithm 1** Thermal aware wake-up schedule algorithm at every node $n_{i}$**INPUT** Node temperature $t_{n_{i}}$ for node $n_{i}$ 1: **loop** 2: At each communication period, $Set,\;\Delta_{t}^{n_{i}}=t_{n_{i}}^{curr}-t_{n_{i}}^{prev}$ 3: **if**
$\Delta_{t}^{n_{i}}>0$ AND $t_{n_{i}}^{curr}<Th_{h}$
**then** 4:  η=min(η×α,maxη) 5: **else if**
$\Delta_{t}^{n_{i}}>0$ AND $t_{n_{i}}^{curr}>Th_{h}$
**then** 6:  η=maxη 7: **else** 8:  η=max(η−β,minη) 9: **end if**10: **end loop**

Initially, every node $n_{i}$ sets η=minη, in particular, nodes exchange their frames in every superframe. At each communication period, a node measures its current temperature, also estimates $\Delta_{t}^{n_{i}}$ which is the difference between the previously estimated temperature and the current temperature. A node multiplicatively increases its communication period with a factor α=2 whenever the temperature increases but still below the hotspot threshold (line 3–4). The communication period is set to the maxη while the temperature reaches $Th_{h}$ (line 5–6). However, a node decreases its communication period additively when the current temperature decreases and $\Delta_{t}^{n_{i}}$ becomes negative or at least no change found from the earlier temperature (line 8).

Overall, Algorithm 1 shows slightly aggressive behavior in raising the communication period while the temperature increases, and in contrast it adopts conservative approach for the decrease of communication period when the node cools down. This is due to considering the thermal increase as the most crucial factor because of its severe consequences to the tissue damage. However, a long communication period also might disrupt the QoS provisioning. Therefore, the constant parameters used in this algorithm need to be chosen carefully that could balance between the QoS provisioning and thermal raise which has been discussed in Section 5.3.1.

### 4.4. MAC Operations

This section introduces the MAC operations of ThMAC during a superframe. As the superframe consists of different periods, we discuss how the frames are exchanged between nodes and the BC for different traffic types in a corresponding period.

#### 4.4.1. Contention Access Period (CAP)

ThMAC exploits CAP for uploading small DATA to the BC. Nodes generating Dc or Nr traffic only contend during this period. Nodes employ the usual CSMA/CA mechanism for data transmission.

Since both the Dc and Nr traffic contend in this period, differentiated CSMA/CA parameters are used to prioritize Dc traffic. Table 1 lists the contention parameters used for both these traffic types. Here, the slot refers to a pCSMASlotLength.

**Figure 6:**
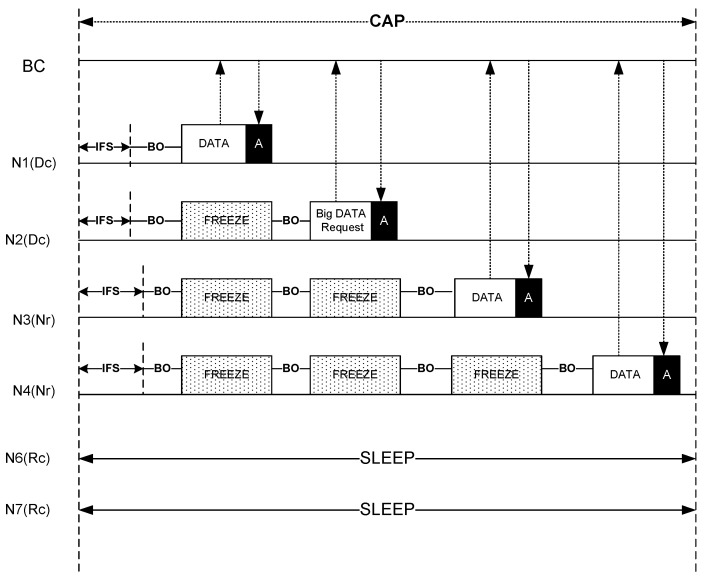
Communication during Contention Access Period (CAP).

Figure 6 illustrates the DATA uploading operation during CAP. During CAP, a Dc traffic source may originate small DATA or request for slot allocation of big DATA. As the figure shows, node N1 originates small Dc DATA and node N2 generates a request for big DATA to the BC. On the other hand, N3 and N4 sources Nr traffic. Due to smaller IFS and Contention Window parameters, both N1 and N2 preempts their transmission over N3 and N4. Nodes having either Dc or Nr traffic continue their operation until the CAP ends. However, during CAP, if any node has traffic other than Dc or Nr, keeps its radio turned off to save energy as well as to prevent temperature increase. In Figure 6, both node N6 and N7 are in sleep state as they possess Rc traffic.

#### 4.4.2. Polling Period

**Figure 7:**
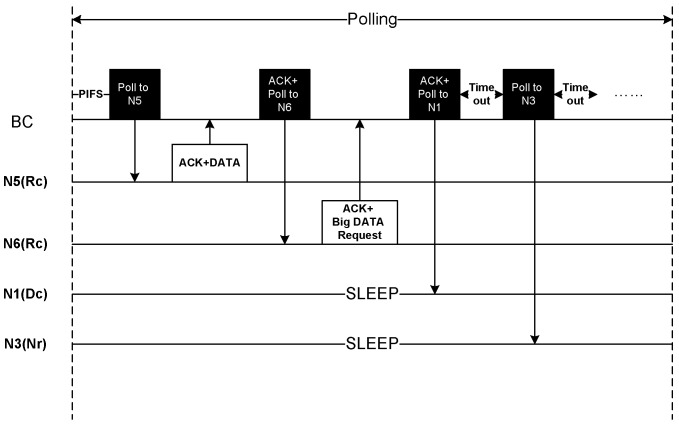
Communication during Polling period.

Figure 7 depicts the Rc traffic transmission for small DATA. We employ polling period for Rc traffic to ensure the reliability as BC performs coordinated transmission with the sensor nodes.

After IFS period, BC starts polling to the sensor nodes. If a node has small Rc DATA it immediately responds with the DATA that serves as an acknowledgement of the poll packet. BC continues transmitting poll packet to the other nodes. The poll packet also acts as an acknowledgement of the earlier DATA packets. A node having big Rc DATA also requests for slot allocation to transmit during CFP period. All the other nodes that do not have Rc traffic remain in the sleep state. As shown in Figure 7, node N5 transmits a small Rc DATA while node N6 appeals for slot allocation for the transmission in the CFP period. Node N1 and N3 are in sleep state due to having Dc and Nr traffic respectively. Therefore, after polling to N1, BC waits for a timeout period and then polls to N3 after the timeout occurs, and continues accordingly.

#### 4.4.3. DL Period

**Figure 8:**
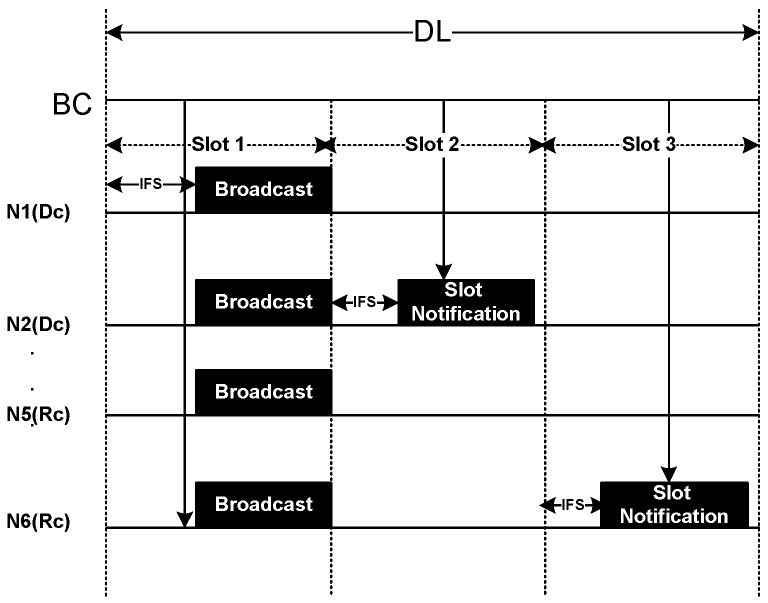
Communication during Download (DL) period.

The DL period is used for download data transmission from the BC to the sensor nodes. BC exploits both broadcast and unicast transmission in this period as depicted in Figure 8. We divide the DL period into a number of slots. Each slot starts with an IFS period followed by download data from BC. In the whole period, all the nodes are in active state. Upon receiving the slot allocation request during CAP or Polling period, BC notifies the respective nodes regarding the slot allocation as unicast packet. As the figure shows, node N2 and N6 having Dc and Rc traffic respectively, receive a slot notification packet from BC in response to their request sent during CAP and Polling period.

#### 4.4.4. CFP Period

**Figure 9:**
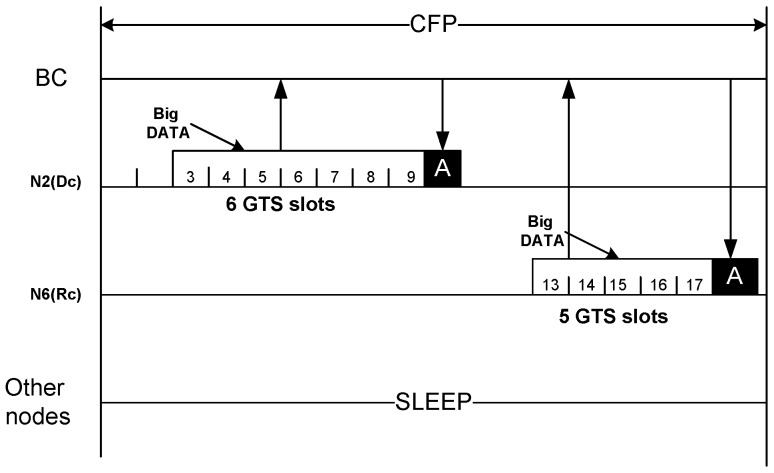
Communication during CFP period.

The CFP period is used only for big DATA transmission from the nodes to the BC. Being notified by BC regarding the assigned slot number, a node wakes up in that particular slot, transmits its big DATA, and then goes to sleep again after receiving the acknowledgement. As the Figure 9 shows, node N2 and N6 transmits their big DATA at their assigned slot no 3 and 13 respectively. Based on the request, N2 has been assigned 6 GTS while N6 is assigned 5 GTS. All other nodes remain in the sleep state.

#### 4.4.5. Emergency Data Management

In a WBAN, emergency traffic can be generated any time when a sudden change in the estimated physiological parameters occur, and needs to be notified to the BC in no time. However, the requirement cannot be fulfilled if a separate period is designated only for Em traffic in a superframe as ThMAC exercises for other traffic types. Thus, we introduce a separate emergency traffic handling mechanism to urgently deliver Em traffic in different periods.

**Figure 10:**
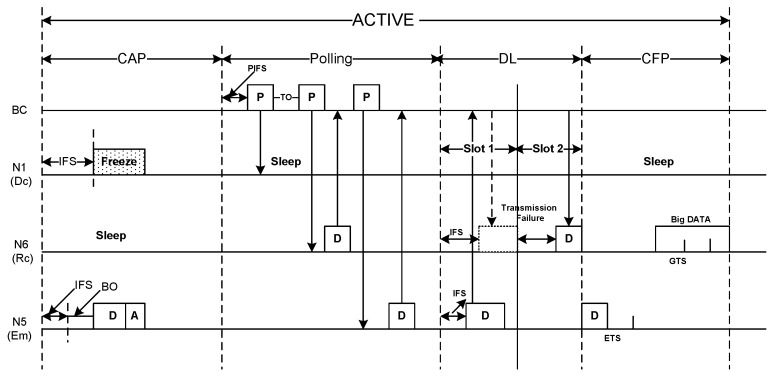
Emergency data transmission during ACTIVE period.

Figure 10 depicts a sample scenario regarding how Em traffic can be transmitted into an ACTIVE period. As explained earlier, CAP in an ACTIVE period is used only for Dc and Nr traffic having diverse contention parameters as listed in Table 1. If a node generates Em traffic during CAP, considering its urgency we choose the contention parameters for Em traffic as follows: IFS = 1 slot, $CW_{min}$ = 2 slots, $CW_{max}$ = 4 slots. As the Figure 10 shows, node N5 having Em traffic preempts N1 having Dc traffic due to lower contention window parameters.

Since BC separately polls every node during polling period, the Em traffic transmission during this period is straightforward. A node just sends its Em traffic in reply of poll packet reception.

As mentioned before, the DL period is for downward data transmission from BC to sensor nodes. However, since Em traffic transmission is in upward direction to BC, ThMAC employs the IFS period for emergency traffic transmission. We adopt the mechanism as described in Reference [16]. At the beginning of every slot BC waits for an IFS period before downloading any data. If a node has emergency traffic, it chooses an IFS period shorter than the IFS chosen by BC, and transmits its emergency data. Thus, the shorter IFS preempts the download data from BC. However, since the preemption causes transmission failure, BC transmits the data in the consecutive slot as depicted in Figure 10.

To transmit Em traffic during CFP, some slots are allocated at the beginning of CFP, known as the Emergency Transmission Slot (ETS). Thus, the remaining slots during CFP are used as GTS for transmitting big DATA. However, since the Em traffic is usually small DATA containing biological parameter, we assume no big DATA will be transmitted as Em traffic. Thus the restriction for transmitting big DATA during CFP is not applied for Em traffic.

**Figure 11:**
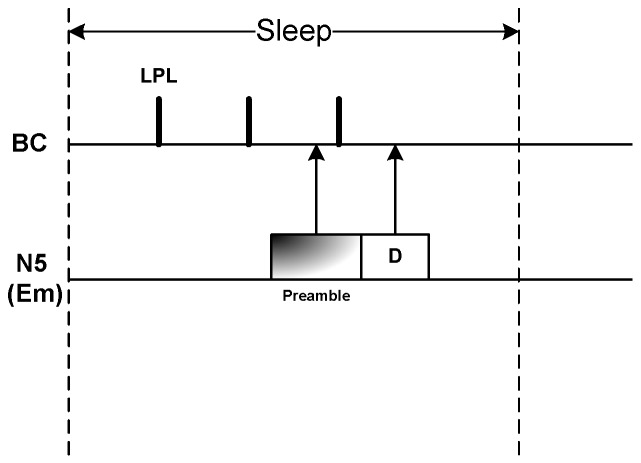
Emergency data transmission during the SLEEP period.

Due to employing duty cycle mechanism as well as thermal-aware wake-up schedule, nodes might remain in long sleep period. However, as Em traffic could be generated during sleep period, we adopt Low Power Listening (LPL) mechanism for BC as described in [28]. Since we assume BC is not power hungry, thus instead of deep sleep, BC performs LPL at certain interval, denoted as $t_{lpl}$. A node having Em traffic during sleep period transmits a long preamble at the duration longer than the sleep period of duty cycle of BC. Sensing the preamble, BC turns into active state and receives the Em data sent by the node right after the preamble as illustrated in Figure 11.

Because of exercising thermal-aware wake-up schedule, a node might sleep between *m* superframes if its communication period is *m*. To prevent thermal raise, we stop the communication activities of the sensor in the midst of an communication period, but the data sensing activity can still be on. However, if a node senses an Em traffic during that period, it immediately wakes-up at the upcoming superframe, receives the beacon, and transmits the Em traffic in the active period of that superframe and then goes to sleep again.

## 5. Performance Evaluation

To evaluate the performance of ThMAC, we conduct experiments through simulations. This section discusses the ThMAC performance evaluation including simulation environment, simulation metrics, and the results obtained.

### 5.1. Simulation Environment

**Figure 12:**
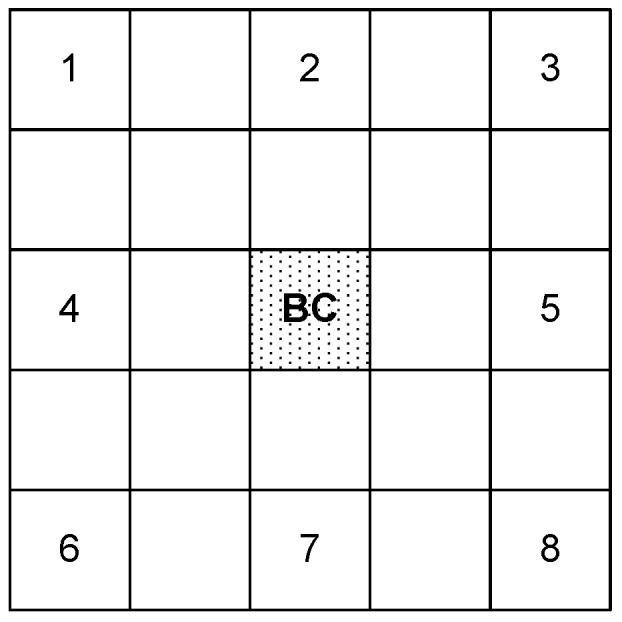
Simulation topology: A 5×5 grid where grid with number represents the sensor ID.

We consider a WBAN with 8 nodes and a BC forming star topology. The 8 nodes are deployed into 5×5 grid as shown in Figure 12. The four traffic types are distributed to the nodes as depicted in Table 2. The simulation program has been developed in C++.

Table 3 shows the detailed simulation parameters including physical layer, MAC layer and the parameters related with thermal rise estimation. Throughout the simulation, we ignore the effect of bit error rate (BER). Hence, packet drop occurs only due to collision and buffer overflow. The initial data generation of the nodes is randomized. Dc, Rc and Nr traffic are generated periodically, where we vary the packet generation rate from 0.5 packet per second (pps) to 4 pps in each experiment. We generate 10% packet as big DATA of total generated packets for each of Dc and Rc traffic type. We randomize the Em traffic generation throughout the simulation period. We compare the performance of ThMAC with IEEE 802.15.6 standard with beacon mode as it is the baseline protocol for WBAN. In IEEE 802.15.6 standard, we designate Exclusive Access Phase (EAP) for transmitting Em (mapped to user priority 7), and Dc traffic (mapped to priority 6), Managed Access Phase (MAP) for Rc traffic (mapped to priority 3), and Contention Access Phase (CAP) for transmitting Nr traffic (mapped to priority 0). Each simulation is carried out for 100 s and the average values are obtained over 10 random runs.

### 5.2. Performance Metrics

We used the following metrics to evaluate ThMAC.

*Maximum Temperature Rise*. Maximum temperature rise denotes the maximal estimated temperature by any node throughout the simulation period. The metric manifests the protocol performance for hotspot formation and the associated tissue damage.

*Average Temperature Rise*. The average temperature rise of the nodes denotes the average temperature increase of all the nodes from their initial temperature.

*Energy Consumption.* The energy consumption, *E*, is estimated as

(4) $$E=\sum_{i=1}^{n}P_{l}T_{l}^{i}+P_{t}T_{t}^{i}+P_{r}T_{r}^{i}+P_{s}T_{s}^{i}$$

where *n* is the number of nodes, $P_{l}$, $P_{t}$, $P_{r}$, $P_{s}$ denote the listening power, transmission power, reception power and sleep power, and $T_{l}^{i}$, $T_{t}^{i}$, $T_{r}^{i}$ and $T_{s}^{i}$ refer to the time spent in listening, transmitting, receiving and sleeping state respectively.

*End-to-End Latency.* End-to-End latency of a packet is estimated as the difference between the packet generation time and the time the packet is received by the BC. Latency experienced by distinct data packets are averaged over the total number of distinct packets received by BC.

*Packet delivery ratio (PDR).* It is the ratio of the total number of unique packets received by BC to the total number of packets generated by the nodes.

### 5.3. Simulation Results

This section discusses the results obtained through different simulation experiments.

#### 5.3.1. Impact of Constant Parameters

The constant parameter values used in Algorithm 1 are empirically chosen. We observe the impact of different values of those parameters. The parameter values are selected in such a way that could balance the thermal rise as well as QoS provisioning, particularly, in terms of latency. In this experiment, we choose the data generation rate of Dc, Rc and Nr traffic as 2 pps, and Em traffic is randomly generated. The value of α and β is set to 2 and 1 respectively.

**Figure 13:**
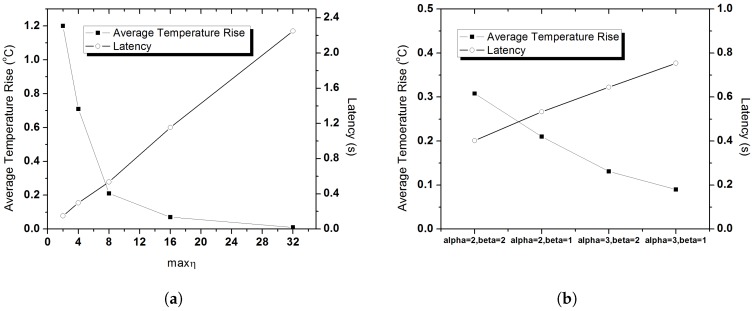
Impact of constant parameters. (**a**) Impact of maxη on thermal rise and latency. (**b**) Impact of α,β on thermal rise and latency.

Figure 13a illustrates the impact of maxη on average temperature rise as well as latency. Since we opt to maintain the communication of the nodes at every superframe until the temperature rises, so minη is set to 1. To choose an optimal maxη, we vary its values and observe its impact. As the figure shows, with the increasing maxη value, the average temperature rise falls drastically, and it is obvious due to the long inactive status of the nodes. Contrastively, the latency increases which might affect the QoS demand of the delay constrained nodes. Hence, although a higher maxη value shows better temperature performance, but it inhibits in achieving the desired QoS demands. Therefore, for a better trade off we choose maxη=8, in which the average temperature rise is in moderate level, 0.2 °C, also having lower latency (0.5 s).

The impact of α,β on temperature rise and latency has been depicted in Figure 13b. Here, we set the maxη value as 8. As α is a multiplicative increasing factor and β is an additive decreasing factor, for a bit aggressive α value at 3, and low decreasing factor β=1, the average temperature rise is significantly lower (0.09 °C), yet the average latency is much higher (0.75 s). In contrast, with a bit conservative increasing factor and higher decreasing factor at α=2,β=2, the nodes achieve lower latency although the average temperature rise is higher which is over 0.3 °C. Therefore, we choose α=2,β=1 as an optimal value as it achieves a balanced thermal rise as well as packet latency.

#### 5.3.2. Thermal Performance

**Figure 14:**
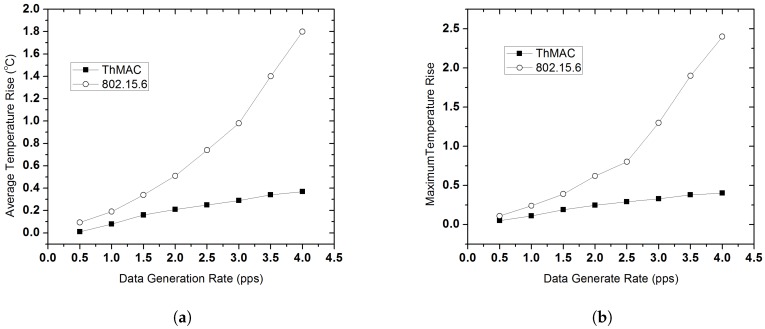
Thermal Performance. (**a**) Average Temperature Rise varying data generation rate. (**b**) Maximum Temperature Rise varying data generation rate.

We evaluate the thermal performance of ThMAC and IEEE 802.15.6 in different data generation rate. Figure 14a shows the average temperature rise for both the protocols in different traffic load. As the traffic load increases, the average temperature also increases for both the protocols. However, due to temperature aware duty cycling mechanism ThMAC depicts significantly better performance compared to 802.15.6. At high traffic load, due to temperature increase, nodes multiplicatively increase the communication period that eventually lowers the average temperature. Contrarily, the lack of any thermal-aware duty cycling mechanism causes a sharp rise in average temperature for 802.15.6.

The maximum temperature rise throughout the simulation period varying data generation rate is depicted in Figure 14b. Even at very high traffic load at 4 pps, the maximum temperature rise ever observed for ThMAC is 0.4 °C which is the hotspot threshold. However, at the same traffic load 802.15.6 shows 2.4 °C thermal rise, which is extremely higher resulting in severe tissue damage.

#### 5.3.3. Latency Performance

**Figure 15:**
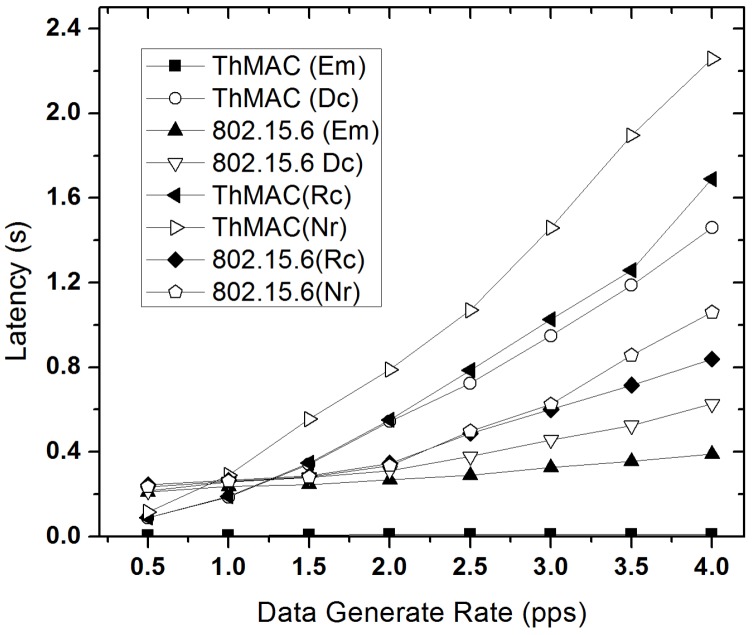
Average latency varying data generation rate.

Figure 15 illustrates the average latency observed for different traffic types for both ThMAC and 802.15.6. Clearly, the increase in traffic load increases the latency. ThMAC significantly outperforms 802.15.6 for delivering emergency traffic due to emergency data management in active period. A remarkably lower latency has been observed for emergency traffic for ThMAC in all the traffic loads. However, as 802.15.6 employs the emergency traffic transmission only in EAP, which is also shared with Dc traffic, the delivery delivery delay increases with the increasing traffic load. Although, at lower traffic load the delay performance for Dc traffic is comparatively lower than that of 802.15.6, but it considerably increases at higher traffic load due to the thermal-aware duty cycle mechanism. However, the disjoint active period allocation of ThMAC for diverse traffic types still causes better delay performance due to lower contention. Similar trend has also been observed for non-delay constrained Rc and Nr traffic. The emergency traffic, on the other hand, is not affected by this duty cycle mechanism as ThMAC allows its transmission in all active periods as well as sleep period.

#### 5.3.4. Reliability Performance

**Figure 16:**
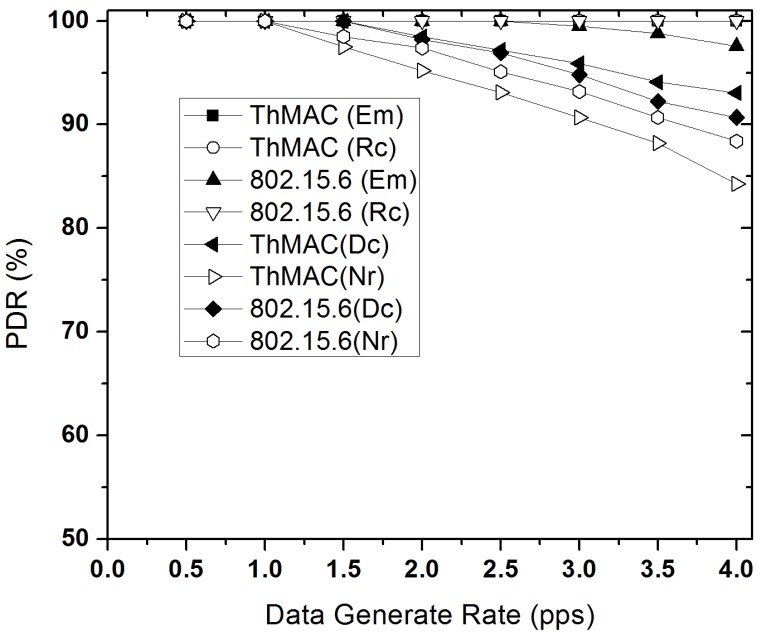
Packet delivery ratio varying data generation rate.

The reliability performance for diverse traffic types of ThMAC is examined for different traffic loads as shown in Figure 16. Having reliability constraints, both Em and Rc traffic achieves 100% PDR in all traffic loads for ThMAC. The emergency data management as well as contention free polling period for Rc traffic causes such better reliability performance. The 10% big DATA is also successfully delivered to the BC in the GTS of the CFP period. Due to employing MAP for Rc traffic 802.15.6 also depicts 100% PDR in all the traffic loads. However, the PDR of Em traffic in 802.15.6 slightly decreases at high traffic load due to the increased contention with Dc traffic during EAP. The non-reliability constrained traffic, Dc and Nr shows a bit poorer reliability performance as the traffic load increases for both ThMAC and 802.15.6.

#### 5.3.5. Energy Performance

**Figure 17:**
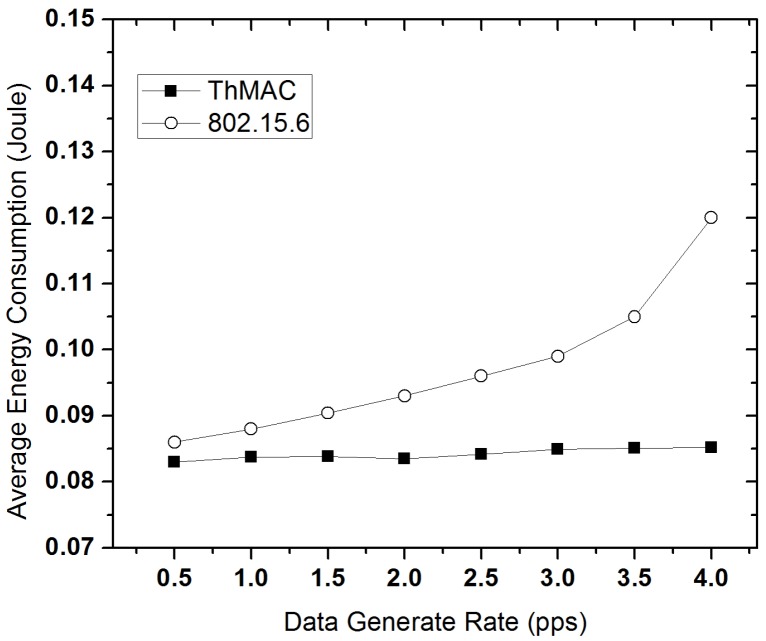
Average energy consumption varying data generation rate.

Figure 17 portrays the average energy consumption for both ThMAC and 802.15.6 in different traffic loads. In all the traffic loads, ThMAC outruns 802.15.6 in terms of energy consumption. As the higher traffic load results in lower duty cycle of the nodes in ThMAC, the average energy consumption does not increase with the increase in data rate. Moreover, the separate transmission period for diverse traffic types causes lower number of packet retransmissions which also influences in achieving lower energy consumption.

#### 5.3.6. Impact of Big DATA

In this experiment, we evaluate the impact of big DATA on the latency and PDR performance of small DATA for both ThMAC and 802.15.6. As the occurrence of big DATA is not common in nature for WBAN, however, its existence might affect the QoS performance of small DATA due to its dominance in the limited active period.

**Figure 18:**
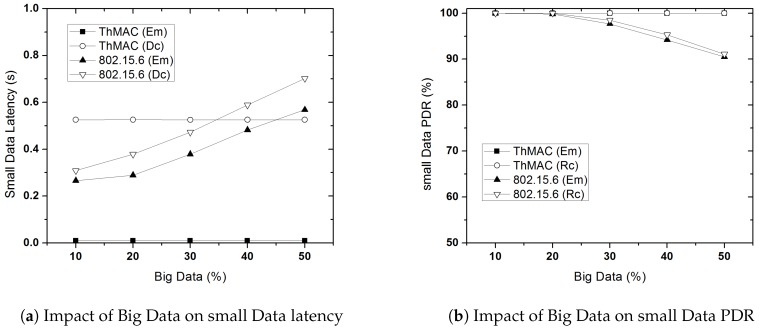
Impact of Big DATA.

Figure 18a illustrates the impact of big DATA on the latency performance of small DATA for both the protocols. Here, we only consider Em and Dc traffic as they are delay constrained. Since ThMAC allocates a distinct contention free period for delivering big DATA, increasing its proportion does not disrupt the latency performance for small DATA. However, with the increasing percentage of big DATA the latency of small DATA considerably increases for both Em and Dc traffic in 802.15.6. This is due to the fact that the big DATA occupies most of the time in EAP period. Thus, in spite of having lower contention window value, Em traffic does not get the channel access for an already on going big DATA transmission. For Dc traffic, the situation is worse than that of Em traffic.

The impact of big DATA on the PDR of small DATA has been depicted in Figure 18b. Here, we show the PDR performance of Em and Rc traffic due to their reliability constraints. The separate CFP for big DATA in ThMAC also does not change the PDR performance of small DATA. However, the proportional increase of big DATA reduces the PDR of Em traffic for 802.15.6. Despite exploiting MAP, the PDR for Rc traffic also decreases while the big DATA percentage is higher. The main reason is the longer occupation time of big DATA which increases node congestion and small DATA loss due to buffer overflow.

## 6. Conclusions

In this paper, we proposed ThMAC-A thermal aware duty cycle MAC protocol for IoT healthcare with the aim of maintaining a bearable temperature level in a body as well as achieving QoS provisioning for diverse traffic types. ThMAC introduces a thermal-aware duty cycle scheme and presents a superframe structure with detailed MAC operations to meet the respective QoS demand of diverse traffic types. We compared the performance of ThMAC with that of the IEEE 802.15.6, a representative standard for WBANs. Simulation results show that ThMAC clearly outperforms IEEE 802.15.6 in achieving lower temperature rise as well as keeping the temperature surrounding a tissue below some hotspot level. ThMAC also achieves to meet the required QoS demands of diverse traffic types in terms of latency and reliability. Moreover, ThMAC has better energy efficiency compared to IEEE 802.15.6, and achieve fairness in QoS provisioning for both small and big DATA.

## Figures and Tables

**Table 1:** Contention parameters.

Dr	Nr
IFS = 2 slots	IFS = 4 slots
$CW_{min}$ = 2 slots	$CW_{min}$ = 8 slots
$CW_{max}$ = 8 slots	$CW_{max}$ = 16 slots

**Table 2:** Traffic Distribution.

Traffic Type	Sensor ID
Em	1,8
Dc	3,6
Rc	4,5
Nr	2,7

**Table 3:** Parameters and their values used in the simulation.

Type	Parameter	Value
Physical Layer	Transmission band	2.4 GHz
	Data Rate	250 kbps
	Tx Power	2.428 mW
	Rx Power	1.814 mW
	Sleep Power	0.027 mW
	Listening power	1.814 mW
	PHY Header	6 bytes
	Channel Encoding Ratio	2
MAC Layer	Beacon length	10 bytes
	ACK length	8 bytes
	Poll length	7 bytes
	small Payload length	7 bytes
	Big Payload length	10–50 bytes
	$T_{B}$	500 ms
	pCSMASlotLength	40 μs
	GTS Slot time	448 μs
	CAP	20 ms
	Polling period	15 ms
	DL period	10 ms
	CFP	55 ms
	Sleep period	410 ms
	$t_{lpl}$	1 ms
	Long Preamble length	950 μs
	Tx Range	5 m
	Sensing range	10 m
	Max Retry Limit	3
	Queue Size	10 packets
Thermal Rise	Relative Permittivity at 2 MHz, ϵ	826
	Conductivity at 2 MHz, σ	0.5476 $\left[\frac{S}{m}\right]$
	$P_{c}$	0.002
	$C_{p}$	3600 $\left[\frac{J}{\mathrm{kg}{}^{{}^{\circ}}C}\right]$
	Blood perfusion constant, *b*	2700 $\left[\frac{J}{m^{3}s{}^{{}^{\circ}}C}\right]$
	Discretized Time step, $\Delta_{t}$	500 ms
	$T_{b}$	37 °C
	Current provided to sensor antenna, *I*	0.1 A
	Mass density, ρ	1040 $\frac{\mathrm{kg}}{m^{3}}$
	*K*	0.498 $\left[\frac{J}{\mathrm{ms}{}^{{}^{\circ}}C}\right]$
	Discretized Space Step, Δ	0.2 m
	Hotspot threshold, $Th_{h}$	37.4 °C
